# An individualized prognostic signature and multi-omics distinction for early stage hepatocellular carcinoma patients with surgical resection

**DOI:** 10.18632/oncotarget.8212

**Published:** 2016-03-19

**Authors:** Lu Ao, Xuekun Song, Xiangyu Li, Mengsha Tong, You Guo, Jing Li, Hongdong Li, Hao Cai, Mengyao Li, Qingzhou Guan, Haidan Yan, Zheng Guo

**Affiliations:** ^1^ College of Bioinformatics Science and Technology, Harbin Medical University, Harbin 150086, China; ^2^ Key Laboratory of Ministry of Education for Gastrointestinal Cancer, Department of Bioinformatics, School of Basic Medical Sciences, Fujian Medical University, Fuzhou 350001, China

**Keywords:** hepatocellular carcinoma, relative expression orderings, gene expression, prognostic signature, multi-omics

## Abstract

Previously reported prognostic signatures for predicting the prognoses of postsurgical hepatocellular carcinoma (HCC) patients are commonly based on predefined risk scores, which are hardly applicable to samples measured by different laboratories. To solve this problem, using gene expression profiles of 170 stage I/II HCC samples, we identified a prognostic signature consisting of 20 gene pairs whose within-sample relative expression orderings (REOs) could robustly predict the disease-free survival and overall survival of HCC patients. This REOs-based prognostic signature was validated in two independent datasets. Functional enrichment analysis showed that the patients with high-risk of recurrence were characterized by the activations of pathways related to cell proliferation and tumor microenvironment, whereas the low-risk patients were characterized by the activations of various metabolism pathways. We further investigated the distinct epigenomic and genomic characteristics of the two prognostic groups using The Cancer Genome Atlas samples with multi-omics data. Epigenetic analysis showed that the transcriptional differences between the two prognostic groups were significantly concordant with DNA methylation alternations. The signaling network analysis identified several key genes (e.g. TP53, MYC) with epigenomic or genomic alternations driving poor prognoses of HCC patients. These results help us understand the multi-omics mechanisms determining the outcomes of HCC patients.

## INTRODUCTION

Liver cancer is the third-leading cause of death from cancer and over 90% of primary liver cancers are hepatocellular carcinoma (HCC) [1]. The first-line treatment option for HCC patients with well preserved liver function is resection but approximately 60%–70% patients will suffer from recurrence in 5 years [2–4], due to either intrahepatic metastases or the development of de novo tumors [4]. Recurrence is the main causative factor for the poor prognosis of HCC patients [5]. However, the currently used clinical and pathologic features for recurrence risk prediction, such as TNM stage, hepatitis B virus or hepatitis C virus and cirrhosis, are incapable to provide accurate evaluation. Therefore, it is urgent to develop an accurate molecular signature for predicting postsurgical patients with high risk of recurrence.

Many researchers have tried to establish prognostic signatures based on gene expression profiles for HCC patients [6–12]. However, all the previously reported prognostic signatures were based on predefined risk threshold values summarized from expression measurements of the signature genes in the training datasets. Such signatures cannot be directly applied to independent datasets because gene expression profiles are vulnerable to systematic measurement biases due to the notorious experimental batch effects [13, 14]. Although many data normalization methods have been proposed to correct such biases, they can hardly achieve the goal and even distort the real biological signals [15, 16]. Besides, data normalization requires collection of samples beforehand and the risk stratification of patients depends on the heterogeneous risk composition of the samples adopted for normalization together [16, 17]. This could produce substantial uncertainty for patient risk stratification and be impractical for clinical applications [16, 17]. An efficient solution would be to take full advantage of the within-sample relative expression orderings (REOs) of genes, which are robust to batch effects and resistant to monotonic data transformation [14, 18, 19]. As demonstrated in our previous studies for breast cancer [19, 20] and lung cancer [21], prognostic signatures based on within-sample REOs can directly and robustly analyze individual disease samples, in a one-by-one manner, measured by different laboratories. Another problem of previously reported prognostic signatures of HCC is that many of them are generated in patients with advanced stage [10, 12, 22] or regardless of stage [8, 9], which might be less relevant to the prognosis of resectable HCC. Therefore, it is worthy employing the REOs-based method to identify robust prognostic signatures for early stage HCC.

In this study, using three datasets of gene expression profiles for HCC patients, we developed and validated a REOs-based prognostic signature consisting of 20 gene pairs. In the validation dataset of the HCC samples from The Cancer Genome Atlas (TCGA) [23], we used the TCGA multi-omics data to analyze the distinct epigenomic and genomic characteristics of two prognostic groups. Moreover, the signaling network analysis further revealed several key genes with epigenomic or genomic alternations determining the outcomes of HCC patients.

## RESULTS

### Development and validation of the REOs-based prognostic signature

**Figure 1:**
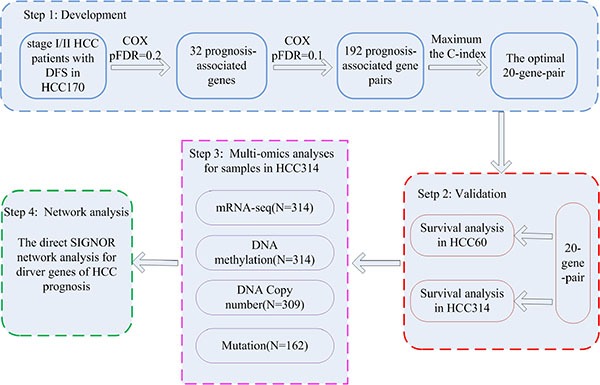
The workflow for construction and validation of the prognostic signature The workflow showed four major analysis steps: the development (step 1) and validation (step 2) of the gene pairs signature; multi-omics characteristics analyses of distinct prognostic groups (step 3) and the SIGNOR network analysis for HCC prognostic genes (step 4).

**Figure 2:**
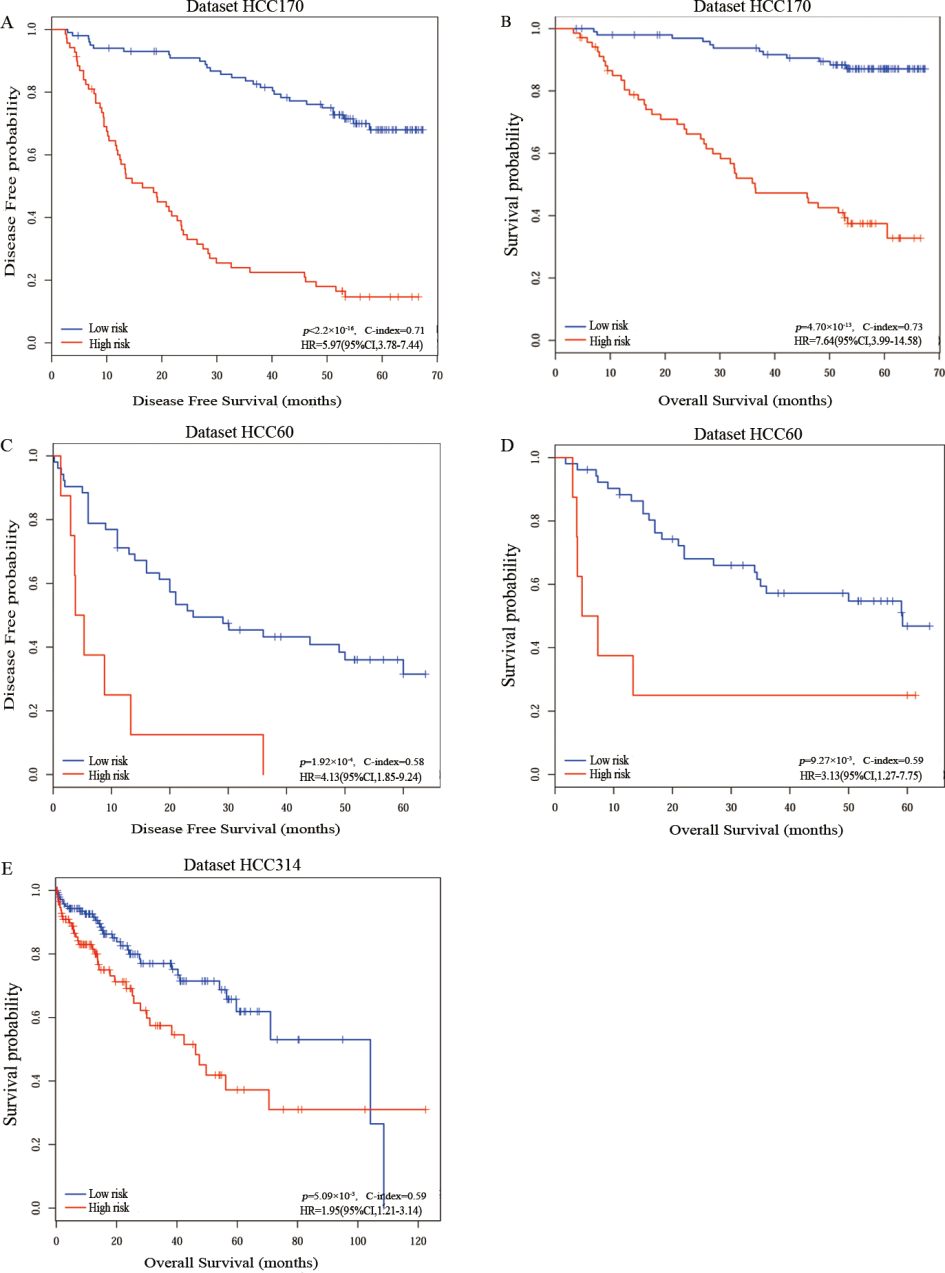
The Kaplan-Meier curves of disease-free survival and overall survival for prognostic groups predicted by the 20-gene-pair in the training and validation datasets Kaplan-Meier curves of disease-free survival (**A**) and overall survival (**B**) for the training dataset HCC170; Kaplan-Meier curves of disease-free survival (**C**) and overall survival (**D**) for the validation dataset HCC60; Kaplan-Meier curves of overall survival (**E**) for the validation dataset HCC314. A sample was classified into the high-risk group (red line) if and only if at least 10 of the 20 prognostic gene pairs voted for high-risk; otherwise, the low-risk group (blue line).

Our general flowchart was described in Figure 1. Using the gene expression profile of 170 HCC samples measured by the GPL3921 platform (Table 1), denoted as HCC170, we found 32 genes whose expression levels were significantly correlated with the disease-free survival (DFS) of HCC patients (univariate Cox proportional-hazards regression model, pFDR < 20%). For every two of the 32 prognosis-associated genes, according to their REOs in each sample, we classified all samples into two subgroups and evaluated whether the two subgroups of samples had significantly different DFS. Totally 192 prognosis-associated gene pairs were identified (univariate Cox regression model, pFDR < 10%). The 20 gene pairs with the highest C-index values (C-index = 0.71) were selected as the final prognostic signature (Table 2) and patients were classified into the high-risk group when at least ten gene pairs suggested that this patient was at high risk (see Materials and Methods and Figure 1). According to this rule, the 170 samples in the training dataset were stratified into a low-risk group with 101 samples and a high-risk group with 69 samples, and the former group had significantly better DFS (HR = 5.97, 95% CI: 3.78–9.44, *p* < 2.2 × 10^−16^, C-index = 0.71, Figure 2A) and overall survival (OS) (HR = 7.64, 95% CI: 3.99–14.58, *p* = 4.70 × 10^−13^, C-index = 0.73, Figure 2B) than the latter group. A multivariate COX regression analysis showed that the 20-gene-pair prognostic signature remained significantly associated with patients’ DFS after adjusting for TNM stage, hepatitis B virus infection, liver cirrhosis and α-fetoprotein, as shown in Table 3.

**Table 1:** Description of the datasets used in this study

	HCC170	HCC60		HCC314
**Accession**	GSE14520	GSE14520	E-TABM-36	TCGA
**Platform**	GPL3921	GPL571		IlluminaHiseq-RNAseqV2
**Sample Size**	170	21	39	314
**Age**	51.5 (21–77)	47 (33–76)	68 (18–79)	61 (18–90)
**Median follow-up period(month)**	53.4 (3.3–67.4)	34.4 (1.8–63.8)	35 (3–60)	10.93 (0–122.5)
**Gender**				
Male	143 (84%)	20 (95%)	31 (79%)	219 (70%)
Female	27 (16%)	1 (5%)	8 (21%)	95 (30%)
**TNM Stage**				
I	93	3	0	154
II	77	1	0	71
III	0	2	0	65
IV	0	0	0	4
NA	0	15	39	20
**HBV**				
AVR-CC	46	2	0	0
CC	119	4	0	0
NA	5	15	39	314
**Cirrhosis**				
yes	153	20	0	6
no	17	1	0	3
NA	0	0	39	305
**AFP**				
> 300 ng/mL	66	10	0	56
< = 300 ng/mL	101	10	0	178
NA	3	1	39	80

Abbreviations: NA, not available; AVR-CC, active viral replication chronic carrier; CC, chronic carrier.

**Table 2:** The 20-gene-pair prognostic signature

Signature	Gene A	Gene B	C-index	Beta	pFDR
Pair1	CAT	EBAG9	0.6581	1.19	4.31 × 10^−06^
Pair2	RNF208	MTMR10	0.6121	0.96	9.20 × 10^−05^
Pair3	TUBA8	MTMR10	0.6069	0.92	1.01 × 10^−03^
Pair4	RNF208	GULP1	0.5886	0.79	5.25 × 10^−04^
Pair5	ST18	ADAMTS3	0.5714	0.61	6.10 × 10^−03^
Pair6	OSBPL10	NPM3	0.5679	0.74	2.81 × 10^−03^
Pair7	TUBA8	NPM3	0.5583	0.60	2.06 × 10^−02^
Pair8	TRIM26	TRMT12	0.5565	1.20	2.17 × 10^−04^
Pair9	RNF208	OSBPL10	0.5562	1.33	8.85 × 10^−03^
Pair10	TUBA8	PF4V1	0.5502	1.29	1.68 × 10^−04^
Pair11	ST18	NTS	0.5469	0.49	4.97 × 10^−02^
Pair12	CAT	TRMT12	0.5431	1.74	8.87 × 10^−05^
Pair13	RNF208	SLC52A2	0.5362	1.22	2.82 × 10^−02^
Pair14	SORBS2	HSF1	0.5359	1.34	1.26 × 10^−03^
Pair15	RNF208	HSF1	0.5342	1.97	3.55 × 10^−02^
Pair16	SORBS2	SLC52A2	0.5337	1.82	1.31 × 10^−04^
Pair17	PYGL	EIF3H	0.5333	1.97	3.56 × 10^−02^
Pair18	CAT	NOLC1	0.5326	2.35	3.84 × 10^−05^
Pair19	RNF208	SLC2A1	0.5269	0.70	7.22 × 10^−02^
Pair20	EYA1	PF4V1	0.5264	1.13	7.67 × 10^−03^

Note: Beta the parameters calculated by the univariate Cox regression model. Beta represents the risk coefficient of within-sample REO of gene pair (A, B), where Beta > 0 indicates that *E_a_* < *E_b_* is a risk factor, and vice versa. pFDR, the adjusted *P*-value by storey.

**Table 3:** Univariate and multivariate Cox regression analyses for the 20-gene-pair signature

Variables	Univariate model		Multivariate model	
	HR (95% CI)	*P*	HR (95% CI)	*P*
**HCC170**				
Predictive signature (high vs. low)	5.97 (3.78–9.44)	**< 2.2 × 10^−16^**	5.92 (3.68–9.53)	**2.43 × 10^−13^**
Stage (II vs. I)	2.03 (1.32–3.11)	**9.64 × 10^−4^**	1.62 (1.03–2.54)	**3.66 × 10^−2^**
HBV (AVR-CC vs. CC)	1.41 (0.89–2.22)	0.14	1.14 (0.88–1.81)	0.58
Cirrhosis (yes vs. no)	2.14 (0.87–5.28)	0.09	1.60 (0.63–4.05)	0.32
AFP (> 300 ng/mL vs. < = 300 ng/mL)	0.92 (0.59–1.44)	0.73	0.73 (0.46–1.14)	0.17
HCC314				
Predictive signature (high vs. low)	1.94 (1.21–3.14)	**5.09 × 10^−3^**	1.87 (1.12–3.11)	**1.58 × 10^−2^**
Stage (IV/III vs. II/I)	1.62 (0.91–2.89)	0.10	1.53 (0.85–2.75)	0.16

NOTE: Bold indicates significant *P* values.

Abbreviations: HBV, hepatitis B virus; AVR-CC, active viral replication chronic carrier; CC, chronic carrier.

In the first validation dataset with 60 samples from two different laboratories but measured by the same platform GPL571, denoted as HCC60, 8 and 52 samples were classified into the high- and low-risk groups, respectively. The low-risk group had significantly better DFS (HR = 4.13, 95% CI: 1.85–9.24, *p* = 1.92 × 10^−4^, C-index = 0.58, Figure 2C) and OS (HR = 3.13, 95% CI:1.27–7.75, *p* = 9.27 × 10^−3^, C-index = 0.59, Figure 2D) than the high-risk group. The second validation dataset was composed of 314 TCGA samples of patients with just OS data but no DFS data, denoted as HCC314. The significant correlations between DFS and OS have been reported for gastric cancer [24], colorectal cancer [25], breast cancer [26] and renal cell carcinoma [27]. Here, we also assessed the correlation between DFS and OS in HCC using datasets HCC170 and HCC60. The Pearson's linear correlation coefficients between DFS and OS were 0.78 (95% CI:0.71–0.83) and 0.82 in the two datasets, respectively. The results suggested DFS can be a valid surrogate for OS in HCC. Therefore, for the dataset HCC314, we shifted survival analysis from DFS to OS, which is the golden standard for judging the success of a particular treatment [28]. The low-risk group of 170 patients had a significantly better OS than the high-risk group of 144 patients (HR = 1.95, 95% CI:1.21–3.14, *p* = 5.09 × 10^−3^, C-index = 0.59, Figure 2E). Due to the lack of clinical parameters for many patients in the two validation datasets, we only analyzed whether the 20-gene-pair prognostic signature was independent of TNM stage for dataset HCC314. Multivariate COX regression analysis showed that the 20-gene-pair prognostic signature remained significantly associated with patients’ OS after adjusting for TNM stage in dataset HCC314 (Table 3).

Further, we were able to provide evidence that the 20-gene-pair prognostic signature was independent of stage. From the HCC170 dataset, we detected 1, 212 and 1, 074 differentially expressed genes (DEGs) (Student's *t*-test, FDR < 10%) between the high- and low-risk groups for stage I and II patients, respectively. The two DEGs lists shared 287 genes and they all displayed with the same over-/under-expression directions in the high-risk samples compared with the low-risk samples, which was highly unlikely to occur by chance (binomial distribution test, *P* < 2.2 × 10^−16^, see Materials and Methods). Similarly, for the HCC314 dataset, genes shared between any two DEGs lists of the prognostic groups for stage I, II and III were also highly consistent (binomial distribution test, all *P* < 2.2 × 10^−16^) (Supplementary Table S1). These results supported that the 20-gene-pair prognostic signature was independent of stage. In the following text, we analyzed samples in the same dataset together despite of stage.

### Distinct transcriptional and functional characteristics of the prognostic subtypes

Using Student's *t*-test with 1% FDR control, we detected 1, 197 DEGs between the high- and low-risk prognostic groups recognized from the training dataset HCC170. Similarly, 5, 026 DEGs were detected between the high- and low-risk prognostic groups recognized from the validation dataset HCC314. These two lists of DEGs had 684 overlapped genes and the concordance score was 98.10% (binomial distribution test, *P* < 2.2 × 10^−16^, see Materials and Methods). In the validation dataset HCC60 including samples from two different laboratories, we detected 2, 192 DEGs between the two prognostic groups (FDR < 1%) using the Rank Product algorithm which was insensitive to batch effects. This list of DEGs overlapped with 443 of the 1, 197 DEGs detected from the training dataset and the concordance score was 86.46%, which was also unlikely to occur by chance (binomial distribution test, *P* < 2.2 × 10^−16^). These results suggested that the distinct transcriptional characteristics of two prognostic groups recognized from different datasets were highly consistent. The signature genes differentially expressed between the high-risk group and the low-risk group in the three datasets were shown in Supplementary Table S2.

Functional analysis for the 5026 DEGs detected from the HCC314 dataset showed that the genes overexpressed in the high-risk group were significantly enriched in pathways related to cell proliferation and tumor microenvironment, such as cell cycle, NF-kappa B signaling pathway and focal adhesion pathway, whereas the underexpressed genes were significantly enriched in metabolic pathways (hypergeometric distribution model, FDR < 5%, Supplementary Table S3). Thus, compared with the low-risk patients, the high-risk patients might have faster growth and aberrant metabolism associated with poor outcome [23, 29].

### Distinct epigenomic and genomic characteristics of prognostic subtypes

In the HCC314 dataset for TCGA samples, 314 samples had DNA methylation data, which were predicted into 144 high-risk samples and 170 low-risk samples by the 20-gene-pair transcriptional signature, respectively. We identified 855 hypermethylated and 537 hypomethylated genes in the high-risk group compared with the low-risk group (Wilcoxon rank-sum test, FDR < 1%), respectively. Among the 855 hypermethylated genes, 318 were overlapped with the 5, 026 DEGs between the high- and low-risk groups and the concordance score between hypermethylation and underexpression of these overlapped genes was 76.10%, which was unlikely to occur by chance (binomial distribution test, *P* < 2.2 × 10^−16^). Similarly, among the 537 hypomethylated genes, 203 were overlapped with DEGs between the high- and low-risk groups. The concordance score between hypomethylation and overexpression of these overlapped genes was 81.77%, which was also highly unlikely to occur by chance (binomial distribution test, *P* < 2.2 × 10^−16^). These results suggested that epigenetic differences of gene promoters may play an important role in inducing transcriptional difference between the high- and low-risk groups.

In the HCC314 dataset, 309 samples had copy number alteration data, which were stratified into 140 high-risk samples and 169 low-risk samples, respectively. In the high-risk samples, the frequencies of copy number gain at 8q13.3, 8q24.21 were 30.10% and 33.66%, respectively, which were significantly higher than the corresponding frequencies of 25.57% and 29.13% in the low-risk patients (Fisher's exact test, FDR < 10%). Among the 209 genes located in the two amplified regions, 70 genes were included in the list of DEGs between the high-risk group and low-risk group, and all of them were overexpressed in the high-risk group. This was unlikely to occur by chance (binomial distribution test, *P* < 2.2 × 10^−16^). These 70 amplified genes all located at 8q24, supporting previously reported results [30, 31]. 12.30% and 27.18% of the high-risk patients showed copy number loss at 3p13 and 17p11.2, respectively, which were also significantly higher than the corresponding frequencies of 7.12% and 23.31% in the low-risk patients (Fisher's exact test, FDR < 10%). However, the four genes located in the two deleted regions were not included in the list of DEGs between the high- and low-risk groups.

The 162 samples with somatic mutation data in the HCC314 dataset were stratified into 80 high-risk samples and 82 low-risk samples, respectively. We detected 193 genes whose mutation frequencies tended to be different between the two prognostic groups (Fisher's exact test, *P* < 0.05). Impressively, 190 of the 193 genes had higher mutation frequencies in the high-risk group compared with the low-risk group, significantly was unlikely to be observed by chance (binomial test, *P* < 2.2 × 10^−16^). Functional enrichment analysis showed that these 190 mutation genes tended to be enriched in HIF-1 and PI3K-Akt signaling pathways (hypergeometric distribution model, *P* < 0.05) (Supplementary Table S4), suggesting that mutation-induced disturbances of these pathways might increase patients’ survival risk.

### Network analysis of prognosis-associated genes with multi-omic characteristics

**Figure 3:**
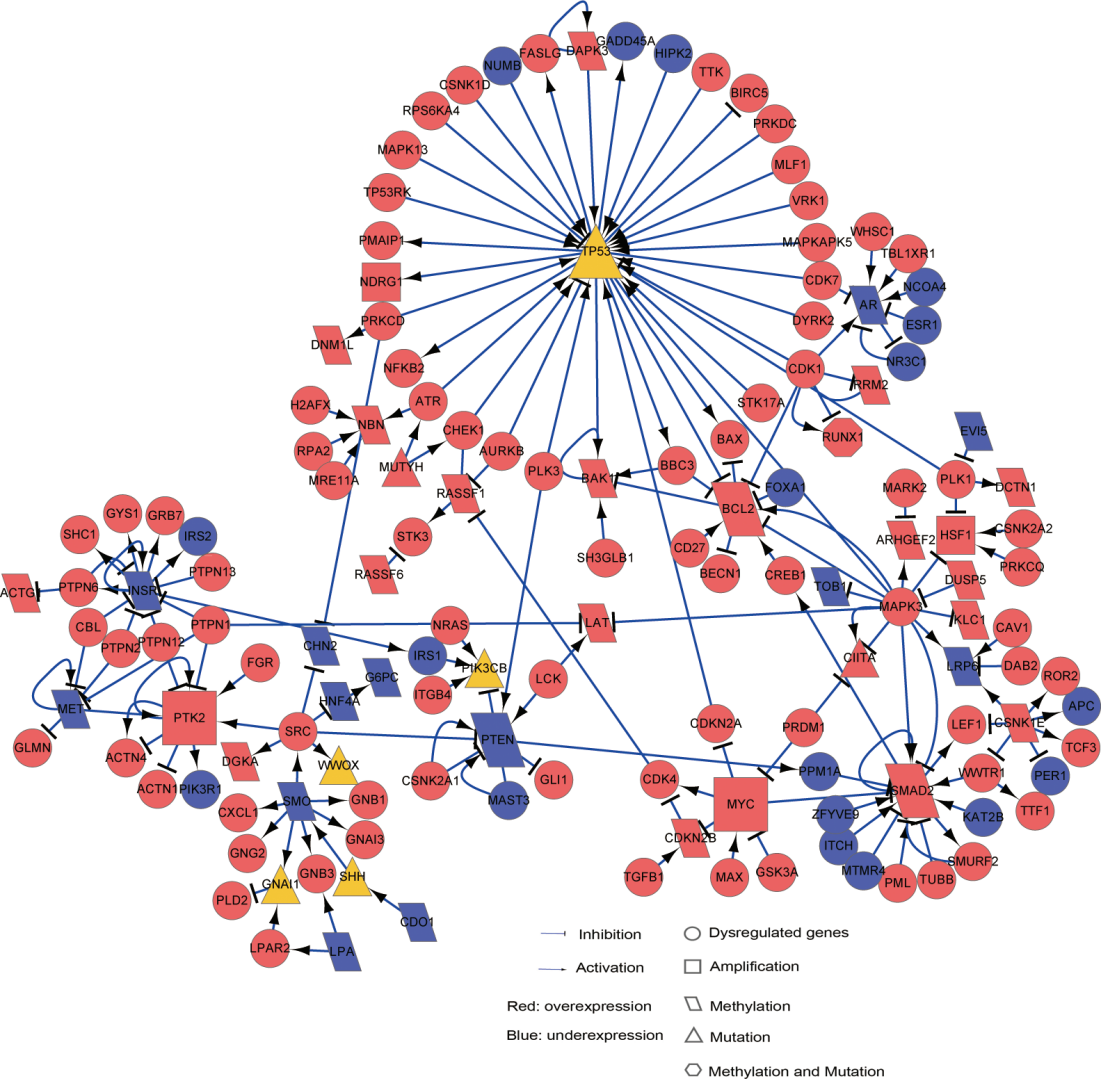
The SIGNOR sub-network for HCC prognosis “drivers” This sub-network included prognosis “drivers”, which have distinct epigenomic and/or genomic alternations with concordant expression changes in the high-risk patients, and other “non-drivers” prognosis-associated DEGs directly linked to the drivers. Circle nodes represent “non-drivers” and the other nodes represent various types of drivers. Rectangle, amplified genes with concordant overexpressions in the high-risk group; Diamond, hypermethylated (or hypomethylated) genes with underexpression (or overexpression) in the high-risk group; Triangle, genes with frequently mutated in the high-risk group; Hexagon, overexpressed genes with hypomethylation and mutation in the high-risk group. The node colors indicate genes’ overexpression (Red) or underexpression (Blue) states in the high-risk group.

We defined “drivers” of the disease prognosis as those genes which had epigenomic and/or genomic alternations with concordant transcriptional changes in the high-risk prognostic group compared with low-risk prognostic group, including 242 hypermethylated and overexpressed genes, 166 hypomethylated and underexpressed genes, 70 amplified and overexpressed genes and 193 mutated genes. Then, based on the activating or inhibitory relations among proteins documented in the SIGnaling Network Open Resource (SIGNOR) [32] database, we constructed a directed network by linking the drivers mapped to SIGNOR with other DEGs which had direct activating or inhibitory links with the drivers. The network included 75 drivers (18 hypermethylated and overexpressed genes, 37 hypomethylated and underexpressed genes, 6 amplified and overexpressed genes and 14 mutated genes) and 133 DEGs directly linked to them (Supplementary Table S5). 208 genes were significantly enriched in 21 biological pathways, including cell cycle, focal adhesion and PI3K-Akt signaling pathway (hypergeometric distribution model, FDR < 10%). The largest connected component (or sub-network) of this network, as shown in Figure 3, included 13 hypermethylated genes, 20 hypomethylated genes, 4 amplified genes and 10 mutated genes in the high-risk group. In the following text, we focused on analyzing six hub genes (TP53, SMAD2, MYC, PTK2, PTEN and BCL2) with the largest degrees (all ≥ 7) in this sub-network.

Three of the six genes, TP53, MYC and SMAD2, were included in the cell cycle pathway. TP53, a tumor suppressor interacting with 41 DEGs in the sub-network, exhibited mutations in 42.50% of the high-risk patients. The mutation of TP53 is correlated with the aggressiveness and poor prognosis of HCC [33, 34]. MYC, interacting with eight DEGs in the sub-network, was overexpressed in the high-risk patients. 74.29% of the high-risk patients carried MYC amplification which can lead to poor prognosis via enhancing cell cycle and proliferation of cancer cell [35]. Besides, MYC had a direct link with SMAD2 which was hypomethylated and overexpressed in the high-risk patients and interacted with 15 DEGs in the sub-network. It is known that the overexpression of SMAD2, a regulator of cell proliferation, apoptosis and differentiation, is correlated with poor survival of gastric carcinoma [36], gliomas [37] and non-small cell lung cancer [38]. Additionally, the overexpression of SMAD2 could be induced by the suppressed activity of PTEN [39], which was hypermethylated and underexpressed in the high-risk patients. It is well known that reduced expression of PTEN is correlated with tumor progression and poor prognosis in HCC patients [40, 41]. As PTEN functions in the focal adhesion pathway, this result suggested a functional interplay between focal adhesion and cell cycle pathways. Another important gene functioning in the focal adhesion pathway, PTK2, was overexpressed in the high-risk patients and amplified together with MYC in 74.29% of the high-risk patients. It has been reported that overexpression of PTK2 is associated with shorter overall survival and higher recurrence rates of HCC [42, 43].

Moreover, the hypomethylated BCL2 in the PI3K-Akt signaling pathway exhibited overexpression in the high-risk patients, which was consistent with the previously reported result [44]. Notably, the perturbed signaling network was closely intertwined, implying that genes involved in different pathways may contribute to poor prognosis of HCC through functional cross-talks. For example, both TP53 mutation and BCL2 overexpression may bring about the development of HCC due to the imbalance between cell proliferation and apoptosis [45].

The above analyses indicated that six hub genes with epigenomic or genomic alternations might play key roles in driving poor prognoses of HCC patients. As only a few genes could be mapped to the sub-network, many prognosis-related pathways were not represented in the sub-network. We will further discuss those prognosis-related pathways in the Discussion section.

## DISCUSSION

In this study, we developed a transcriptional prognostic signature based on within-sample REOs of 20 gene pairs for early stage (I–II) primary HCC tissues. Different from previously reported signatures based on predefined risk scores, the REOs-based prognostic signature is robust against experimental batch effects and data normalization, and can be easily applied at the individual level to samples profiled in different laboratories. Therefore, the REOs-based prognostic signature is a promising type of signature for successfully predicting the DFS and OS in HCC patients after resection. The integrated analyses further revealed the distinct epigenetic and genomic characteristics of the two prognostic groups. The network analysis of prognosis-associated genes with distinct epigenomic or genomic characteristics provides insights into the underlying mechanisms of HCC prognoses.

The genes overexpressed in the high-risk patients were mainly enriched in two groups of pathways. One group was cell proliferation-related pathways, including cell cycle and PI3K-Akt signaling pathway, which were both correlated with poor prognosis of HCC [46–49]. Especially, the cell cycle pathway harbors two key genes (MYC and SMAD2) and the PI3K-Akt signaling pathway includes one key gene (BCL2) which were overexpressed with concordant epigenomic or genomic alternations in the high-risk samples. These results strongly indicated that the high-risk patients’ tumors had higher growth ability than the low-risk patients. Another group of pathways was mainly related to tumor microenvironment, such as focal adhesion and chemokine signaling pathways. Apart from the hub gene PTK2, Osteopontin (OPN) in the focal adhesion pathway, which was overexpressed and hyopmethylated in the high-risk patients, can enhance cell proliferation and metastasis [50] and may be associated with the high recurrence rate and poor survival of HCC after resection [51]. MAPK3 in the chemokine signaling pathway was overexpressed in the high-risk patients. It has been reported that the activation of MAPK3 correlates with poor survival of HCC patients [44]. These results suggested that tumor microenvironment played an important role in the prognosis of HCC patients. A better understanding of the interactions between tumor microenvironment and tumor cells may be helpful for us to identify additional effective treatment targets. For example, sorafenib, a most successful medication of targeted treatment for advanced HCC, can benefit patients’ survival through disrupting the interaction between tumor cells and stromal cells [52, 53].

The genes underexpressed in the high-risk patients were largely enriched in metabolic pathways, including retinol metabolism, carbon metabolism and drug metabolism - cytochrome P450. While it is well known that metabolic pathways are the most frequently disturbed pathways in HCC [53, 54], our results indicated that metabolism functional impairment of liver could induce poor outcome [9, 55, 56]. Besides, HCC is mainly resulted from exposure to external environmental factors which can result in epigenetic changes [29] and consequently would cause changes in gene expression and metabolism [57–59]. The alternation of liver metabolism requires further investigation which may help us better understand the epigenetic processes determining HCC prognoses.

In conclusion, we developed a 20-gene-pair prognostic signature to robustly predict the DFS and OS of postsurgical HCC patients. Further, the multi-omics analyses and the network analysis provided hints on the underlying mechanisms determining the prognoses of HCC patients.

## MATERIALS AND METHODS

### Data sources and data preprocessing

Three datasets of HCC used in this study were downloaded from the Gene Expression Omnibus (GEO, http://www.ncbi.nlm.nih.gov/geo/) [60], ArrayExpress (http://www.ebi.ac.uk/arrayexpress/) [61] and TCGA (http://cancergenome.nih.gov/). From these datasets, we extracted samples with DFS or OS data for analyses, as summarized in Table 1. The GSE14520 [7] dataset included two batches of samples, we used the 170 stage I/II samples from the larger batch to train a prognostic signature. The 21 samples from the smaller batch, together with the 39 samples from the E-TABM-36 [22] dataset, were used as the first validation dataset. The second validation dataset was composed of 314 TCGA samples of patients with only OS data, denoted as HCC314. All samples included in the three datasets were for patients treated with surgery only.

The raw mRNA expression data (.CEL files) for the HCC170 and HCC60 datasets were preprocessed using the Robust Multi-array Average algorithm [62]. Probe-set IDs were mapped to Entrez gene IDs with the corresponding custom CDF files. If multiple probesets were mapped to a gene, the expression value of the gene was defined as the arithmetic mean of the values of the multiple probesets (on the log2 scale).

For the HCC314 dataset, integrative data including level 3 mRNA-seq profiles and DNA methylation profiles and level 2 gene mutation profiles were obtained from TCGA portal. For DNA methylation profiles, we only analyzed the 25, 978 CpG sites located at the promoter regions of genes. Probes that had any “NA”-masked data points and that were designed for sequences on X and Y chromosomes were removed [63]. Probe IDs were mapped to Entrez gene IDs with the corresponding platform file. Totally, 19, 890 CpG sites mapped to 13, 453 genes were analyzed in this study. For gene mutation data, only the non-synonymous mutations were included and a discrete mutation profile including 19, 669 genes were analyzed. Copy number data of level 4 were downloaded from Firehose (https://confluence.broadinstitute.org/display/GDAC/Download). Using the significant regions of gain or loss identified by GISTIC 2.0 [64], we assigned a discrete copy number alteration status to each gene in each sample.

The SIGnaling Network Open Resource (SIGNOR) [32] database, including 3646 proteins with 12, 285 directed relations representing various activating or inhibitory effects, was downloaded and used to constructed a directed gene network.

### Survival analysis

The disease-free survival (DFS) time was defined as the time from surgery to the date of tumor recurrence or distant metastasis, and overall survival (OS) time was defined as the time from surgery to death or the final documented date (censored). The Pearson's linear correlation was used to assess the correlation between DFS and OS. The univariate Cox proportional-hazards regression model was used to evaluate the correlation of expression levels of genes and REOs of gene pairs with patients’ DFS. The multivariate Cox regression model was used to evaluate the independent prognostic value of the signature after adjusting for clinical factors. The C-index proposed by Harrell et al. [65] was used to evaluate the overall concordance between the predicted risk classification and the observed DFS or OS time. Survival curves of DFS and OS between distinct subgroups were visualized with Kaplan-Meier plots and the *p*-value for the difference between the survival curves was calculated by the log-rank tests [66]. The Cox proportional hazards regression model was also used to calculate the hazard ratios (HRs) and their 95% confidence intervals (CIs).

### Identification of prognostic gene pair signature

For a pair of genes, gene A and gene B, all samples were classified into two subgroups according to the REO (*E_a_* > *E_b_* or *E_a_* < *E_b_*) of the gene pair in each sample. Here, *E_a_* and *E_b_* represent the expression levels of gene A and gene B, respectively. We used the univariate Cox proportional-hazards regression model to evaluate whether the patients in the two subgroups had significantly different DFS. The *p*-values were adjusted to positive false discovery rate (pFDR) using the Storey procedure [67], a less stringent and more powerful procedure than the Benjamini and Hochberg FDR control procedure. The significant gene pairs detected with 10% pFDR control were defined as prognosis-associated gene-pairs. Then, the gene pair with the highest C-index was selected as the seed and candidate prognosis-associated gene pairs were added to the signature one at a time until the addition of one gene pair did not improve the C-index. Here, a forward selection procedure was performed to search an optimal subset of the prognosis-associated gene pairs that reached the highest C-index based on the following classification rule: when at least a half gene pairs voting for high risk, a patient was assigned to the high-risk group; otherwise, the low-risk group. The optimal subset of gene pairs with the highest C-index was chosen as the final prognostic gene pair signature. Figure 1 illustrated the flowchart of developing and validating the prognostic signature consisting of gene pairs.

### Analysis of epigenomic and genomic data

The Student's *t*-test was used to select differentially expressed genes (DEGs) between two groups of samples. For the HCC60 dataset including samples from two different laboratories, the Rank Product algorithm [68], which is insensitive to batch effects, was used to select DEGs. The Wilcoxon rank-sum test was used to select differentially methylated (DM) CpG loci between the high- and low-risk samples in the HCC314 dataset. If the promoter of a gene had both hypermethylated and hypomethylated CpG loci, this gene was excluded from sub-sequent analyses [69]. The genes with at least one DM CpG locus were termed DM genes. Fisher's exact test was used to detect genes which had significantly different copy number alternation frequencies or mutation frequencies between two prognostic groups.

### Concordance scores

If two DEGs lists extracted from two independent datasets shared *k* genes, of which *s* genes had the same dysregulation directions (both overexpressed or underexpressed in the two DEGs lists) in the high-risk group compared with the low-risk group, the concordance score was calculated as *s/k*. This score was used to determine the reproducibility of DEGs detected from independent datasets.

If *k* hypermethylated (or hypomethylated) genes were also differentially expressed, of which *s* genes were correspondingly underexpressed (or overexpressed), the concordance score was calculated as *s/k*. This score was used to determine the concordance between DEGs and DM genes.

The cumulative binomial distribution model was used to estimate the probability of observing a concordance score of *s/k* by chance [70]:

$$\begin{array}{l} p=1-\sum_{i=0}^{s-1}\left(\begin{array}{c} k\\ i \end{array}\right){\left(p_{e}\right)}^{i}{\left(1-p_{e}\right)}^{k-i} \end{array}$$

where *p_e_* (here, *P_e_* = 0.5) is the probability of a gene having the concordant relationship between the two gene lists by random chance.

### Pathway enrichment analysis

Functional enrichment analysis was performed based on the Kyoto Encyclopaedia of Genes and Genomes (KEGG) [71]. The hypergeometric distribution model was used to determine biological pathways that were significantly enriched with genes of interest [72]. The Benjamini and Hochberg procedure [73] was used to calculate the False Discovery Rate (FDR). All statistical analyses were done by using the R software package version 3.0.2.

## SUPPLEMENTARY MATERIALS TABLES



## Abbreviations

HCC: hepatocellular carcinoma
REO: relative expression ordering
DFS: disease-free survival
OS: overall survival
BH: Benjamini-Hochberg
DEGs: differentially expressed genes
DM: differentially methylated
C-index: concordance index
FDR: false discovery rate
HRs: hazard ratios
CIs: confidence intervals
AVR-CC: active viral replication chronic carrier
CC: chronic carrier
HBV: hepatitis B virus
