# Supplementary material for: An individualized prognostic signature and multi-omics distinction for early stage hepatocellular carcinoma patients with surgical resection
Source: Oncotarget. 2016 Mar 19;7(17):24097–110. doi: 10.18632/oncotarget.8212 (PMC5029687; doi:10.18632/oncotarget.8212)
Supplement: Supplementary file 1 [file oncotarget-07-24097-s001.pdf]

An individualized prognostic signature and multi-omics distinction for early stage hepatocellular carcinoma patients with surgical resection

Supplementary Materials

Supplementary Table S1: The consistency analysis between any two DEGs lists of the prognostic groups for stage I, II and III in the HCC314 dataset

	No. of DEGs	Comparison	Overlapped	Consistency	Score	*P*-value
Stage I	4661	I VS II	295	295	100%	< 2.2 × E-16
Stage II	492	I VS III	1223	1217	99.51%	< 2.2 × E-16
Stage III	3053	II vs III	234	233	99.57%	< 2.2 × E-16

Note: Differentially expressed genes (DEGs) were identified by Student's *t*-test (FDR < 10%) between the high- and low-risk groups for stage I, II and III patients in the HCC314 dataset, respectively. Concordance scores was calculated as Consistency/Overlapped, *P*-value was used to estimate the probability of observing a concordance score by chance.

Supplementary Table S2: The differential expression of the signature genes in the three datasets

Gene id	Gene symbol	HCC170	HCC60	HCC314
847	CAT	–1	–1	–1
2138	EYA1	1	NA	NA
3297	HSF1	1	NA	1
4922	NTS	1	1	1
5197	PF4V1	1	1	1
5836	PYGL	–1	–1	–1
6513	SLC2A1	1	NA	1
7726	TRIM26	–1	–1	–1
8470	SORBS2	–1	–1	–1
8667	EIF3H	1	–1	1
9166	EBAG9	1	NA	NA
9221	NOLC1	1	NA	1
9508	ADAMTS3	1	1	1
9705	ST18	NA	1	NA
10360	NPM3	1	NA	1
51454	GULP1	1	1	1
51807	TUBA8	–1	NA	NA
54893	MTMR10	1	NA	–1
55039	TRMT12	1	NA	1
79581	SLC52A2	1	NA	1
114884	OSBPL10	1	NA	1
727800	RNF208	–1	NA	NA

Note: Differentially expressed genes (DEGs) between the high-risk group and the low-risk group were identified by Student's *t*-test (FDR < 10%) in the HCC170 and HCC314 datasets. In the validation dataset HCC60 including samples from two different laboratories, DEGs were identified by the Rank Product algorithm (FDR < 10%), which was relatively insensitive to batch effects. 1 and –1 represent genes overexpressed and underexpressed in the high-risk group compared with the low-risk group, respectively; NA represents genes not differentially expressed under the FDR control level of 10%.

Supplementary Table S3: Functional enrichment analysis of overexpressed and underexpressed DEGs in the high-risk group compared with the low-risk group of HCC314 dataset

Pathway Name	FDR
Overexpressed	
Aminoacyl-tRNA biosynthesis	1.39E-03
Ribosome biogenesis in eukaryotes	2.42E-08
Spliceosome	8.56E-07
Chemokine signaling pathway	1.95E-05
NF-kappa B signaling pathway	2.43E-03
Cell cycle	7.06E-11
Phagosome	1.75E-08
Osteoclast differentiation	3.67E-06
Focal adhesion	2.20E-05
ECM-receptor interaction	7.85E-05
Adherens junction	1.61E-03
Platelet activation	1.50E-04
Antigen processing and presentation	2.98E-06
Hematopoietic cell lineage	8.67E-04
Natural killer cell mediated cytotoxicity	7.51E-04
T cell receptor signaling pathway	1.89E-04
Fc gamma R-mediated phagocytosis	1.34E-08
Leukocyte transendothelial migration	1.00E-03
Intestinal immune network for IgA production	2.05E-04
Regulation of actin cytoskeleton	6.07E-05
Vasopressin-regulated water reabsorption	2.05E-04
Underexpressed	
Glycolysis / Gluconeogenesis	1.15E-06
Citrate cycle (TCA cycle)	2.42E-04
Pentose and glucuronate interconversions	8.60E-05
Ascorbate and aldarate metabolism	1.38E-03
Fatty acid elongation	3.79E-03
Fatty acid degradation	2.20E-16
Synthesis and degradation of ketone bodies	6.29E-04
Steroid biosynthesis	7.87E-03
Primary bile acid biosynthesis	4.80E-07
Caffeine metabolism	3.44E-04
Alanine, aspartate and glutamate metabolism	2.84E-04
Glycine, serine and threonine metabolism	1.94E-12
Valine, leucine and isoleucine degradation	2.20E-16
Lysine biosynthesis	8.65E-03
Lysine degradation	8.32E-06
Arginine and proline metabolism	2.03E-07
Histidine metabolism	3.66E-08
Tyrosine metabolism	5.21E-06
Phenylalanine metabolism	4.43E-03
Tryptophan metabolism	2.69E-08

Phenylalanine, tyrosine and tryptophan biosynthesis	6.95E-03
beta-Alanine metabolism	1.11E-07
Starch and sucrose metabolism	4.85E-05
Glycerolipid metabolism	3.70E-04
Pyruvate metabolism	6.46E-08
Glyoxylate and dicarboxylate metabolism	6.24E-08
Propanoate metabolism	4.51E-12
Butanoate metabolism	5.67E-11
One carbon pool by folate	5.97E-03
Retinol metabolism	7.49E-12
Metabolism of xenobiotics by cytochrome P450	4.09E-06
Drug metabolism - cytochrome P450	1.13E-09
Drug metabolism - other enzymes	1.18E-04
Biosynthesis of unsaturated fatty acids	2.12E-03
Carbon metabolism	1.66E-10
Fatty acid metabolism	6.32E-07
Biosynthesis of amino acids	8.81E-04
ABC transporters	1.18E-04
PPAR signaling pathway	2.45E-08
Peroxisome	2.20E-16
Complement and coagulation cascades	1.97E-12
Circadian rhythm	4.94E-03
Fat digestion and absorption	9.70E-04
Bile secretion	7.19E-05

FDR(< 10%) was adjusted by Benjamini and Hochberg.

Supplementary Table S4: Functional enrichment analysis of 190 genes with higher mutation frequencies in the high-risk group compared with the low-risk group in HCC314 dataset

Pathway Name	*P*-value
Caffeine metabolism	4.84E-02
Inositol phosphate metabolism	2.10E-02
Biotin metabolism	2.93E-02
Base excision repair	4.15E-02
HIF-1 signaling pathway	1.95E-02
PI3K-Akt signaling pathway	4.59E-02
Focal adhesion	4.97E-02
Platelet activation	3.69E-02

Supplementary Table S5: DEGs in the SIGNOR sub-network the SIGNOR

Symbol	Over-expression	Under-expression	Hyper-methylation	Hypo-methylation	Amplifi-cation	Mutation
ABL1	TRUE					
ACTG1	TRUE			TRUE		
ACTN1	TRUE					
ACTN4	TRUE					
AGO2	TRUE				TRUE	
AKT3	TRUE					
APC		TRUE				
APH1B	TRUE			TRUE		
APOE		TRUE	TRUE			
AR		TRUE	TRUE			
ARHGEF18	TRUE					
ARHGEF2	TRUE			TRUE		
ATR	TRUE					
AURKB	TRUE					
BAK1	TRUE			TRUE		
BAX	TRUE					
BBC3	TRUE					
BCL2	TRUE			TRUE		
BECN1	TRUE					
BIRC5	TRUE					
BTK	TRUE					
CAV1	TRUE					
CBL	TRUE					
CD27	TRUE					
CDC14B		TRUE				
CDC25B	TRUE					
CDCA3	TRUE			TRUE		
CDK1	TRUE					
CDK4	TRUE					
CDK7	TRUE					
CDKN2A	TRUE					
CDKN2B	TRUE			TRUE		
CDO1		TRUE	TRUE			
CHEK1	TRUE					
CHN2		TRUE	TRUE			
CIITA	TRUE					TRUE
CREB1	TRUE					
CSF1	TRUE					
CSF3R	TRUE			TRUE		
CSNK1D	TRUE					
CSNK1E	TRUE			TRUE		
CSNK2A1	TRUE					
CSNK2A2	TRUE					

CXCL1	TRUE					
CXCL8	TRUE					
DAB2	TRUE					
DAPK3	TRUE			TRUE		
DCTN1	TRUE			TRUE		
DGKA	TRUE			TRUE		
DNM1L	TRUE			TRUE		
DUSP5	TRUE			TRUE		
DYRK2	TRUE					
ELF4	TRUE			TRUE		
ESR1		TRUE				
EVI5		TRUE	TRUE			
F2		TRUE	TRUE			
F2RL2	TRUE			TRUE		
FASLG	TRUE					
FGR	TRUE					
FOXA1		TRUE				
G6PC		TRUE	TRUE			
GADD45A		TRUE				
GLI1	TRUE					
GLMN	TRUE					
GNAI1						TRUE
GNAI3	TRUE					
GNB1	TRUE					
GNB3	TRUE					
GNG2	TRUE					
GRB2	TRUE					
GRB7	TRUE					
GSK3A	TRUE					
GYS1	TRUE					
H2AFX	TRUE					
HCLS1	TRUE			TRUE		
HDAC7	TRUE					
HIPK2		TRUE				
HNF4A		TRUE	TRUE			
HSF1	TRUE				TRUE	
IFNG	TRUE					
IFNGR2	TRUE			TRUE		
IGF1		TRUE	TRUE			
IKZF1	TRUE					
IL22	TRUE					
IL22RA1		TRUE	TRUE			
INSR		TRUE	TRUE			
IRS1		TRUE				
IRS2		TRUE				
ITCH		TRUE				

ITGAV	TRUE					
ITGB4	TRUE					
KAT2B		TRUE				
KDR		TRUE				
KLC1	TRUE			TRUE		
KSR1	TRUE					
LAT	TRUE			TRUE		
LCK	TRUE					
LCP2	TRUE					
LEF1	TRUE					
LPA		TRUE	TRUE			
LPAR2	TRUE					
LRP6		TRUE	TRUE			
LYN	TRUE			TRUE		
LYZ	TRUE					
MAP3K1	TRUE					
MAP3K7						TRUE
MAP4K1	TRUE			TRUE		
MAPK13	TRUE					
MAPK3	TRUE					
MAPKAPK5	TRUE					
MARK2	TRUE					
MAST3		TRUE				
MAX	TRUE					
MCM10	TRUE					
MELK	TRUE					TRUE
MET		TRUE	TRUE			
MLF1	TRUE					
MRE11A	TRUE					
MTMR4		TRUE				
MUTYH	TRUE					TRUE
MYC	TRUE				TRUE	
NBN	TRUE			TRUE		
NCOA4		TRUE				
NDRG1	TRUE				TRUE	
NEK2	TRUE					
NFKB2	TRUE					
NR1I3		TRUE	TRUE			
NR3C1		TRUE				
NRAS	TRUE					
NUMB		TRUE				
OPN	TRUE			TRUE		
PER1		TRUE				
PIK3CB						TRUE
PIK3R1		TRUE				
PLD2	TRUE					

PLK1	TRUE					
PLK3	TRUE					
PMAIP1	TRUE					
PML	TRUE					
POU2AF1	TRUE					
POU2F2	TRUE			TRUE		
PPM1A		TRUE				
PPP1CC	TRUE			TRUE		
PPP1R15A	TRUE					
PPP3CB	TRUE					
PRDM1	TRUE					
PRKCD	TRUE					
PRKCQ	TRUE					
PRKDC	TRUE					
PSENEN	TRUE					
PTEN		TRUE	TRUE			
PTK2	TRUE				TRUE	
PTPN1	TRUE					
PTPN12	TRUE					
PTPN13	TRUE					
PTPN2	TRUE					
PTPN3		TRUE				
PTPN6	TRUE					
RAC1	TRUE					
RAC2	TRUE					
RALGDS	TRUE					
RASSF1	TRUE			TRUE		
RASSF6	TRUE			TRUE		
RECQL4	TRUE				TRUE	
RIN1	TRUE			TRUE		
RIT1	TRUE			TRUE		
ROR2	TRUE					
RPA2	TRUE					
RPS6KA4	TRUE					
RRM2	TRUE			TRUE		
RUNX1	TRUE			TRUE		TRUE
RXRA		TRUE				
SEPT9				TRUE		
SH3GLB1	TRUE					
SHC1	TRUE					
SHH						TRUE
SLAMF1	TRUE					
SMAD2	TRUE			TRUE		
SMARCC1	TRUE					
SMARCE1	TRUE					TRUE
SMO		TRUE	TRUE			

SMURF2	TRUE					
SORL1		TRUE				
SRC	TRUE					
SRGAP3						TRUE
STK17A	TRUE					
STK3	TRUE					
SYK	TRUE					
TBL1XR1	TRUE					
TCF3	TRUE					
TGFB1	TRUE					
TGFB2	TRUE			TRUE		
TGFB3	TRUE			TRUE		
TOB1		TRUE	TRUE			
TP53						TRUE
TP53RK	TRUE					
TRAF1	TRUE			TRUE		
TRAF2	TRUE					
TTF1	TRUE					TRUE
TTK	TRUE					
TUBB	TRUE					
VEGFC						TRUE
VRK1	TRUE					
WASF1	TRUE					
WHSC1	TRUE					
WWOX						TRUE
WWTR1	TRUE					
ZFYVE9		TRUE				
